# Bipolar disorders in ICD-11: current status and strengths

**DOI:** 10.1186/s40345-019-0165-9

**Published:** 2020-01-20

**Authors:** Jules Angst, Vladeta Ajdacic-Gross, Wulf Rössler

**Affiliations:** 1 Department of Psychiatry, Psychotherapy and Psychosomatics, Psychiatric Hospital, University of Zurich, Zurich, Switzerland; 2 Institute of Psychiatry, Laboratory of Neuroscience (LIM 27), University of São Paulo, São Paulo, Brazil; 3 Department of Psychiatry and Psychotherapy, Charité University Medicine, Berlin, Germany

**Keywords:** Bipolar, ICD-10, ICD-11, DSM-5

## Abstract

**Background:**

The Clinical descriptions and diagnostic guidelines for the ICD-11 Classification of mental and behavioural disorders should soon be finalized. To measure their potential impact, the new proposed definitions of bipolar disorders in ICD-11 were applied to data from the Zurich cohort study and compared with the definitions of ICD-10 and DSM-5.

**Results:**

We found little difference between ICD-11 and ICD-10 in the identification of subjects with bipolar disorders, but compared to DSM-5 a considerable increase in the diagnosis of hypomanic episodes and therefore of bipolar-II disorders.

**Conclusions:**

Compared to ICD-10 and DSM-5 the definition of hypomanic episodes according to ICD-11 represents important progress. A higher prevalence of BP-II disorder makes sense from a clinical point of view. Further transcultural research is needed into whether out-patient treatment should be included as a criterion for hypomania. Pure mania is unfortunately missing as an independent and codable disorder in the international diagnostic manuals, whether ICD-11 or DSM-5.

## Introduction

The World Health Organization is central to the continuous development of the diagnostic classification of medical disorders. With the ICD-11 officially approved, the next stage in our field will be the publication of the Clinical Descriptions and Diagnostic Guidelines (CDDG) for the manual’s Mental and Behavioural Disorders section. The guidelines are currently out for consultation and comment. From 2022 the ICD-11 should be in worldwide use. The ICD review process has taken over ten years, partly overlapping with the fifth revision of the DSM of the American Psychiatric Association, which was completed in 2013. From the outset the general aim was to harmonise their structure and descriptions in order to avoid arbitrary differences (Reed et al. 2019).

Differences persist nonetheless. In its conception the ICD-11 is guided by the principles of clinical usefulness and global applicability. In particular, its descriptions of the “essential features” of mood episodes are not presented as equivalents of strict diagnostic criteria. Symptom counts or duration cut-offs are generally avoided, which is designed to reflect clinical practice and the exercise of clinical judgment. A full account of the changes in ICD-11 and their rationale is to be found in (Reed et al. 2019).

The purpose of this short paper is to compare the definitions of manic and hypomanic episodes in ICD-11 (as it currently stands) (a) briefly with the previous version ICD-10 (World Health Organization 1992) and (b) with DSM-5 of the American Psychiatric Association (American Psychiatric Association 2013). By applying the criteria to data from the Zurich study, described in detail in an earlier publication (Angst et al. 2016), we can provisionally illustrate some consequences of the revisions.

### Zurich study sample and definitions

The Zurich study is an epidemiological study, which followed the probands over a period of 30 years from the ages of 19/20 to 49/50. The interview sample was selected from an initial cohort of 4547 people (f = 2346; m = 2201) representative of the canton of Zurich in Switzerland, who were screened in 1978 by the Symptom Checklist 90 (SCL-90-R) (Derogatis 1977) when they were 19 years old (males) or 20 years old (females).

To increase the probability of the development of psychiatric syndromes, a stratified sub-sample of 591 subjects was selected for the longitudinal study. Two-thirds of the sample consisted of high scorers (defined as scoring above the 85th percentile of the global severity index of the SCL-90-R) (n = 396) and one-third of a random sample of those with scores below the 85th percentile (n = 195). The use of stratified samples is not uncommon in epidemiological research (Dunn et al. 1999). Altogether, seven interview waves were conducted: in 1979, 1981, 1986, 1988, 1993, 1999 and 2008. For the present analysis only data collected from 1986 onwards were used; in 1986 an interview section on mania was added to the interview. The initial allocation to the two strata did not change over the study’s time span.

The analysis is presented both with the raw figures (n = 475 in the period 1986–2008) and with figures after reweighting to offset the stratification (n = 2051). For the reweighting, the analysis was conducted with the SAS procedure PROC SURVEYFREQ.

For the definitions of ICD-11 we have used the documents available on the internet. For the ICD-10, diagnostic criteria for research were subsequently published, distinct from of the CDDG for clinicians, (ICD-10 DCR) (World Health Organization 1993). These imposed both symptom numbers and duration cut-offs, narrowing the differences between the ICD and DSM-IV. We applied ICD-10 DCR definitions in our analyses of ICD-10 but this approach was not extended to ICD-11, as there is no indication that diagnostic research criteria will be produced for this latest revision.

## Bipolar disorders in ICD-11

The mood disorders section in ICD-11 has been reorganized, opening with the description of mood episodes (depressive, manic, mixed and hypomanic), which are not coded. Codes are ascribed to disorders, which are diagnosed on the basis of the pattern of a patient’s mood episodes over time. Moreover, in the bipolar and related disorders grouping newer research led to the subdivision of bipolar disorder into types I and II, based on the distinction between mania and hypomania (Reed et al. 2019). This is in line with DSM-5.

### Mania as a diagnosis

Historically bipolar disorders (BP) included mania, BP-I, BP-II, hypomania and cyclothymic disorders. The international diagnostic manuals do not reflect the full affective spectrum. In none of them does mania have the status of a separate disorder. Since ICD-10 a manic episode has been codable only within mood disorders. Those with mania/a manic episode are diagnosed as having bipolar-I disorder. ICD-11 continues this tradition, which is in line with the successive versions of the DSM, including DSM-5. This must nonetheless be considered a loss in both clinical and research terms. There is growing evidence from epidemiological, clinical and genetic studies that unipolar mania exists as a distinct disorder (Merikangas et al. 2014; Baek et al. 2014; Angst and Grobler 2015). A recent analysis of data merged from seven epidemiological studies of adults found BP-I (mania with major depressive disorder) in 323 subjects and pure mania in 109, a relatively rare but separate diagnosis (Angst et al. 2018).

### Major change in the definitions of manic and hypomanic syndromes and episodes in ICD-11 and DSM-5

The definitions of manic and hypomanic syndromes and episodes in the two manuals are now almost identical as regards entry criteria, duration, hospitalization, and the presence or absence of psychotic features and impairment in social and occupational functioning, albeit with the dissimilarities referred to above (essential features vs. lettered entry criteria, etc.).

All manic and hypomanic episodes in ICD-11 require as defining features:

1. euphoria, irritability or expansiveness, and, increased activity or subjective experience of increased energy
2. plus “several “(ICD-11), three or more (DSM 5) of the following 7 symptoms:
  1. increased talkativeness or pressured speech,
  2. flight of ideas,
  3. increased self-esteem or grandiosity,
  4. decreased need of sleep,
  5. distractibility,
  6. impulsive reckless behaviour,
  7. increase in sexual drive, sociability or goal-directed activity.

Thus, a diagnosis of bipolar mood disorders in both ICD-11 and DSM-5 now requires as an essential entry feature not only the presence of elated/euphoric, expansive or irritable mood but in addition, in all cases, increased activity/energy; this is in contrast to both ICD-10 and DSM-IV TR (American Psychiatric Association 2000) which required as criterion A only the presence of mood changes. This development is not without problems. Earlier, we illustrated the consequences of this important conceptual change, as introduced by DSM-5, with data from the Bridge Study. 810 of 3618 (22.5%) patients with Major Depression manifested only one mood criterion for bipolarity (elated or irritable). Following DSM-IV criteria those patients were diagnosed with bipolar disorders, whereas according to DSM-5 they lost their bipolarity and were re-diagnosed as having major depressive disorder (Angst et al. 2014).

### Manic and hypomanic episodes in ICD-11 compared to DSM-5

DSM-5 defines manic episodes and hypomanic episodes slightly more restrictively (as had the ICD-10 DCR) in that for cases with mood irritability only (without euphoria) it requires 4 rather than 3 of the 7 symptoms for a diagnosis; ICD-11 does not single out irritability in this way.

Similarly the minimum duration of a hypomanic episode is 4+ days in DSM-5 and “several days” in ICD-11 For the purposes of our analysis several was interpreted as 4+ consecutive days.

One further restriction in the definition of a hypomanic episode in DSM-5 relates to changes in functioning. In DSM-5 criterion C: an unequivocal change in functioning uncharacteristic of the person, and criterion D: the disturbance in mood and the change in functioning are observable by others must be met. In ICD-11 this is less categorically expressed as the significant change in the usual range of moods and behaviour would be apparent to people who know the individual well.

### Exclusions

Like DSM-5, ICD-11 excludes syndromes caused by the effects of a substance or medication, another medical condition (tumour etc.), but now allows the diagnosis of a manic or hypomanic episode if the full syndrome persists after antidepressant treatment (e.g. medication, ECT, light therapy) is discontinued and its direct effects have ceased or receded.

Causal attributions and exclusions are, however, known to be problematic and not data based.

## Comparison of diagnostic systems using data from the Zurich study: ICD-10, ICD-11 and DSM-5

### ICD-11 and ICD-10: manic symptoms, hypomania and mania

Comparing ICD-11 with ICD-10 (ICD-10 DCR) our analysis (Table 1) found a minimal reduction in the frequency of ICD-11 manic episodes (N = 29) compared to ICD-10 (31). On the other hand ICD-11 diagnosed 48 subjects with a hypomanic episode vs. 40 by ICD-10.

**Table 1:** ICD-11 vs ICD-10: manic spectrum including hospitalization lifetime

	ICD-11 Mania	Hypomania	Manic symptoms	Others	Total
ICD-10 mania	28	1	2	0	31
Hypomania	0	40	0	0	40
Manic symptoms	1	7	144	0	152
Others	0	0	0	252	252
Total	29	48	146	252	475

Prevalence rates have to be weighted, because in the Zurich study risk cases (high scorers on the SCL-90 R) were purposely over-represented (Table 2).

**Table 2:** ICD-11 vs. ICD-10: prevalence rates, weighted (%) with 95% confidence intervals

	ICD-11 Mania	Hypomania	Manic symptoms	Others	Total
ICD-10 Mania	5.4 (2.4–8.4)	0.05 (0.0–0.1)	0.6 (0.0–1.7)	0	6.0 (2.8–9.2)
Hypomania	0	8.5 (4.7–12.3)	0	0	8.5 (4.7–12.3)
Manic symptoms	0.05 (0.0–0.1)	1.3 (0.0–2.9)	27.1 (21.2–33.1)	0	28.5 (22.5–34.6)
Others	0	0	0	57.0 (50.3–63.7)	57.0 (50.3–63.7)
Total	5.4 (2.4 –8.5)	9.9 (5.8–13.9)	27.7 (21.7–33.7)	57.0 (21.7–33.7)	100

### ICD-11 and DSM-5: manic symptoms, hypomania and mania

ICD-11 and DSM-5 are in close agreement in the diagnosis of manic episodes (Table 3) but hypomanic episodes are far more frequently identified by ICD-11 (N = 48) than DSM-5 (N = 15); only 10 subjects are classified by both.

**Table 3:** ICD-11 vs DSM-5: manic spectrum including hospitalization lifetime

	ICD-11 mania	Hypomania	Manic symptoms	Others	Total
DSM-5 mania	23	0	2	0	25
Hypomania	1	10	4	0	15
Manic symptoms	5	37	140	0	182
Others	0	1	0	252	253
Total	29	48	146	252	475

Application of ICD-11 results in prevalence of mania in 5.4%, DSM-5 in 4.7%. But hypomania is diagnosed by ICD-11 far more frequently in 9.9% compared to DSM-5 in 2.2%. The prevalence rates are shown in Table 4.

**Table 4:** ICD-11 vs. DSM-5: prevalence rates, weighted (%) with 95% confidence intervals

	ICD-11 mania	Hypomania	Manic symptoms	Others	Total
DSM-5 mania	4.1 (1.5–6.8)	0	0.6 (0.0–1.7)	0	4.7 (1.9–7.6)
Hypomania	0.05 (0.0–0.1)	1.5 (3.0–15.1)	0.7 (0.0–1.8)	0	2.2 (0.3–4.1)
Manic symptoms	1.2 (0.0–2.8)	8.3 (4.6–12.1)	26.4 (20.5–32.3)	0	36.0 (29.5–42.7)
Others	0	0.05 (0.0–0.1)	0	57.0 (50.3–63.7)	57.0 (50.4–63.7)
Total	5.4 (2.4–8.5)	9.9 (5.8–13.9)	27.7 (21.7–33.7)	57.0 (50.3–63.7)	100

## Validity of the ICD-11 diagnoses

As validators we can provide family history (parents, siblings) and lifetime treatment for manic or depressive symptoms.

As is clear in Table 5, a positive family history (FH+) is strongly correlated with the diagnostic groups of mania and hypomania. Both diagnostic subgroups correlate significantly in their FH + for mania, suicide/attempts, alcohol abuse/dependence and anxiety/panic. But there is no association with a FH + for depression or smoking.

**Table 5:** ICD-11 mania: family history (parents and siblings)

ICD-11	Mania (29) %	Hypomania (48) %	Manic symptoms (146) %	Others (252) %	Chi square P
Family history					
Mania	24.1	18.7	8.9	3.2	< .0001
Depression	62.1	58.3	57.5	49.6	< .284
Suicidality	37.9	12.5	18.5	16.3	< .025
Anxiety/panic	58.6	47.9	32.2	24.6	< .0001
Alcohol abuse	17.2	12.5	24.7	11.5	< .0061
Smoking	72.4	72.3	79.3	71.1	< .389

Probands’ responses only; consent to interview relatives was not given

Table 6 lists percentage treatment rates for manic and depressive symptoms for ICD-11, ICD-10 and DSM-5. The three diagnostic classifications show very similar validity regarding the treatment of manic symptoms but none for the treatment of depressive symptoms.

**Table 6:** Lifetime treatment for manic or depressive symptoms

	Mania	Hypomania	Manic symptoms	P
%Treatment manic symptoms				
ICD.11	26.7	12.5	2.7^a^	.0001
ICD-10	25.8	15.0	2.6	.0001
DSM-5	32.0	13.3	4.4	.0001
% Treatment depr. symptoms				
ICD.11	70.0	56.2	53.7	.259
ICD-10	67.7	57.5	53.9	.365
DSM-5	64.0	60.0	54.9	.663

^a^4 of 149 subjects were treated as out-patients

If ICD currently classifies patients who have been hospitalized for manic syndromes as having manic episodes, individuals with manic symptoms who have been treated could reasonably be diagnosed as hypomanic. In Table 6 we show this to be the case for 4 of 149 subjects (2.7%), who, if added to the subjects with hypomania, would increase their number from 48 to 52. The prevalence rate would correspondingly increase from 9.9 to 10.1%. Of course further research into this approach is needed.

In order to test the hypothesis of a continuum from normal to pathological, one might also look at recurrent brief hypomania among those with manic symptoms (Angst 1997). It is present in 37 subjects, with a prevalence of 7.8%. Cumulatively 23.38% of individuals manifested manic, hypomanic or brief hypomanic episodes.

## Conclusions

Compared to ICD-10 and DSM-5, the current ICD-11 definition of hypomanic episodes represents important progress. Hypomanic episodes will be diagnosed twice as frequently as manic episodes. From a clinical point of view it makes sense that the milder condition should occur more often than the severe one. This also means that bipolar-II disorder will become a more common diagnosis. It should be remembered that hypomanic episodes in terms of their social and subjective consequences are not always undesirable. They can be associated with an increased work capacity and heightened creativity. Transcultural research is needed into whether out-patient treatment should be included as a criterion for a diagnosis of hypomania, in parallel with the hospitalization criterion for a manic episode. Finally, ICD-11 allows greater scope for the exercise of clinical judgment, which is an important strength of this classification system.

## Data Availability

Not available.
